# The extracellular matrix in peripheral nerve repair and regeneration: a narrative review of its role and therapeutic potential

**DOI:** 10.3389/fnana.2025.1628081

**Published:** 2025-10-21

**Authors:** Miriam Metafune, Luisa Muratori, Federica Fregnan, Giulia Ronchi, Stefania Raimondo

**Affiliations:** Department of Clinical and Biological Sciences and Neuroscience Institute Cavalieri Ottolenghi (NICO), University of Torino, Orbassano, Italy

**Keywords:** extracellular matrix, nerve injury, nerve regeneration, therapeutic approaches, hydrogel, conduits, decellularized nerves

## Abstract

The extracellular matrix (ECM) is a non-cellular and gelatinous component of tissues, rich in proteins and proteoglycans, that provides information about the environment, forms a reservoir of trophic factors and regulates cell behavior by binding and activating cell surface receptors. This important network acts as a scaffold for tissues and organs throughout the body, playing an essential role in their structural and functional integrity. It is essential for cells to connect and communicate with each other and play an active role in intracellular signaling. Due to these properties, in recent decades the potential of the extracellular matrix in tissue engineering has begun to be explored with the aim of developing innovative biomaterials to be used in regenerative medicine. This review will first outline the components of the extracellular matrix in the peripheral nerve, followed by an exploration of its role in the regeneration process after injury, with a focus on the mechanisms underlying its interactions with nerve cells. Qualitative and quantitative methods used for extracellular matrix analysis will be described, and finally an overview will be given of recent advances in nerve repair strategies that exploit the potential of the extracellular matrix to enhance regeneration, highlighting the critical issues of extracellular matrix molecule use and proposing new directions for future research.

## 1 Introduction

The extracellular matrix (ECM) is the most complex structural component of tissues, consisting of proteins and polysaccharides that form a tightly organized network. ECM is a dynamic structure that is in constant synthesis and degradation, mediated by several matrix-degrading enzymes during normal and pathological conditions. It functions as a scaffold for tissues, providing biological support and attachment for cells while connecting either to ECM-producing cells or to those nearby. The ECM significantly influences cellular activities, such as adhesion, migration, proliferation, differentiation, cell survival, and homeostasis, during physiological and pathological processes through various integrin and non-integrin cell surface receptors (Gao et al., 2013; Gonzalez-Perez et al., 2013). Additionally, ECM components sequester growth factors as well as molecules like water and minerals.

**Figure 1:**
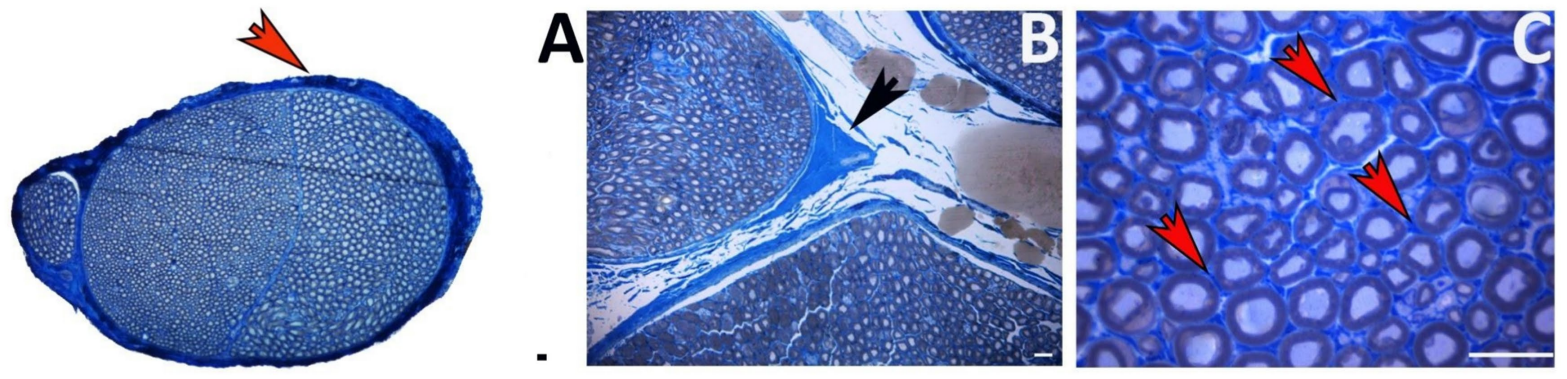
Toluidine blue-stained transverse section of a healthy nerve showing the ECM localization. **(A)** Epineurium around the whole nerve (red arrow). **(B)** Perineurium among nerve fascicles (black arrow). **(C)** Endoneurium among nerve fibers (red arrows). Scale bar = 20 μm.

Anatomically, the ECM in a peripheral nerve is distributed throughout the three layers of connective tissue: epineurium, perineurium, and endoneurium (Figure 1). The epineurium, the outermost layer, surrounds the whole nerve (Figure 1A). It consists of loose connective tissue that houses the blood vessels supplying the nerve (Gao et al., 2013; Gonzalez-Perez et al., 2013). Nerve fascicles are surrounded by perineurium, a thin but dense sheath composed of flat perineurial cells and an outer layer of collagen fibers organized in bundles (Figure 1B). The supporting connective tissue that covers each nerve fiber is the endoneurium (Figure 1C), organized in endoneurial tubes comprising fibroblasts, ECM components, and basal lamina arranged in continuity around the axon-Schwann cells (SC) units (Gonzalez-Perez et al., 2013).

**Figure 2:**
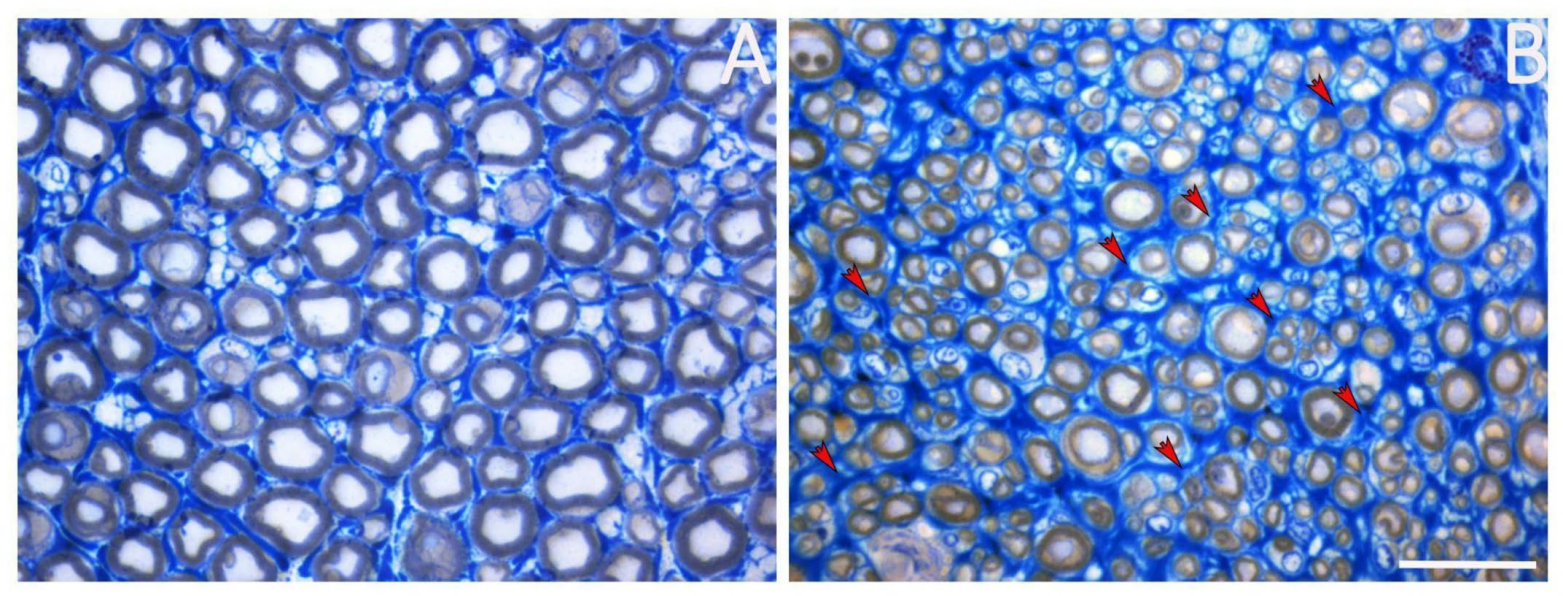
Representative image of a healthy nerve **(A)** and a regenerated nerve **(B)**. Red arrows mark the presence of microfascicles detectable within the regenerated nerve. Scale bar = 20 μm.

The presence of ECM in peripheral nerves is crucial, as it plays a key role in regulating various biological mechanisms, both during nerve development and throughout the regeneration process following injury. Indeed, after a nerve injury, several mechanisms occur at the injury site almost immediately, including morphological and metabolic changes and ECM remodeling (Gantus et al., 2006): axons distal to the lesion site are disconnected from the neuronal body and undergo “Wallerian degeneration,” a process characterized by axonal degeneration, myelin degradation, and macrophage recruitment. During these steps, endoneurial tubes are shrunk and filled by proliferating SCs and macrophages (Lee and Wolfe, 2000). Throughout the regenerative process, SCs basal lamina persists, allowing SCs alignment to create Büngner bands, which provide a supportive and growth-promoting microenvironment for axonal elongation; at the same time, ECM remodeling takes place, creating microfascicles of regenerated nerve fibers, typically detectable within a regenerated nerve (Figure 2). SCs also provide specific cues for axonal regeneration (Stoll et al., 1989), and their behavior is strongly affected by ECM components. Gantus et al. (2006) pointed out that during nerve regeneration, some ECM components, such as laminin, fibronectin, and type IV collagen, are able to support axonal growth and elongation; other ECM molecules, such as chondroitin sulfate proteoglycans, can inhibit axonal outgrowth (Zuo et al., 1998). The balance between positive and negative signals from ECM molecules to SC generates a specific response that either promotes or inhibits axonal regrowth (Stevens and Jacobs, 2002). As will be discussed in this review, the success of peripheral nerve regeneration is associated with changes in the ECM components and receptors (Gantus et al., 2006).

This review aims to provide a thorough understanding of the ECM in peripheral nerve repair and regeneration, emphasizing its biological roles, therapeutic potential, and future directions. It covers the composition of the ECM in peripheral nerves and the receptors and signaling pathways that facilitate communication between the ECM and nerve cells. The dynamic role of the ECM during nerve regeneration is explored, alongside the main methods used to study these processes. The review also highlights innovative repair strategies that harness ECM components to promote regeneration and discusses the challenges and opportunities that lie ahead for ECM-based therapies in peripheral nerve repair.

## 2 Extracellular matrix composition in peripheral nerves

**Figure 3:**
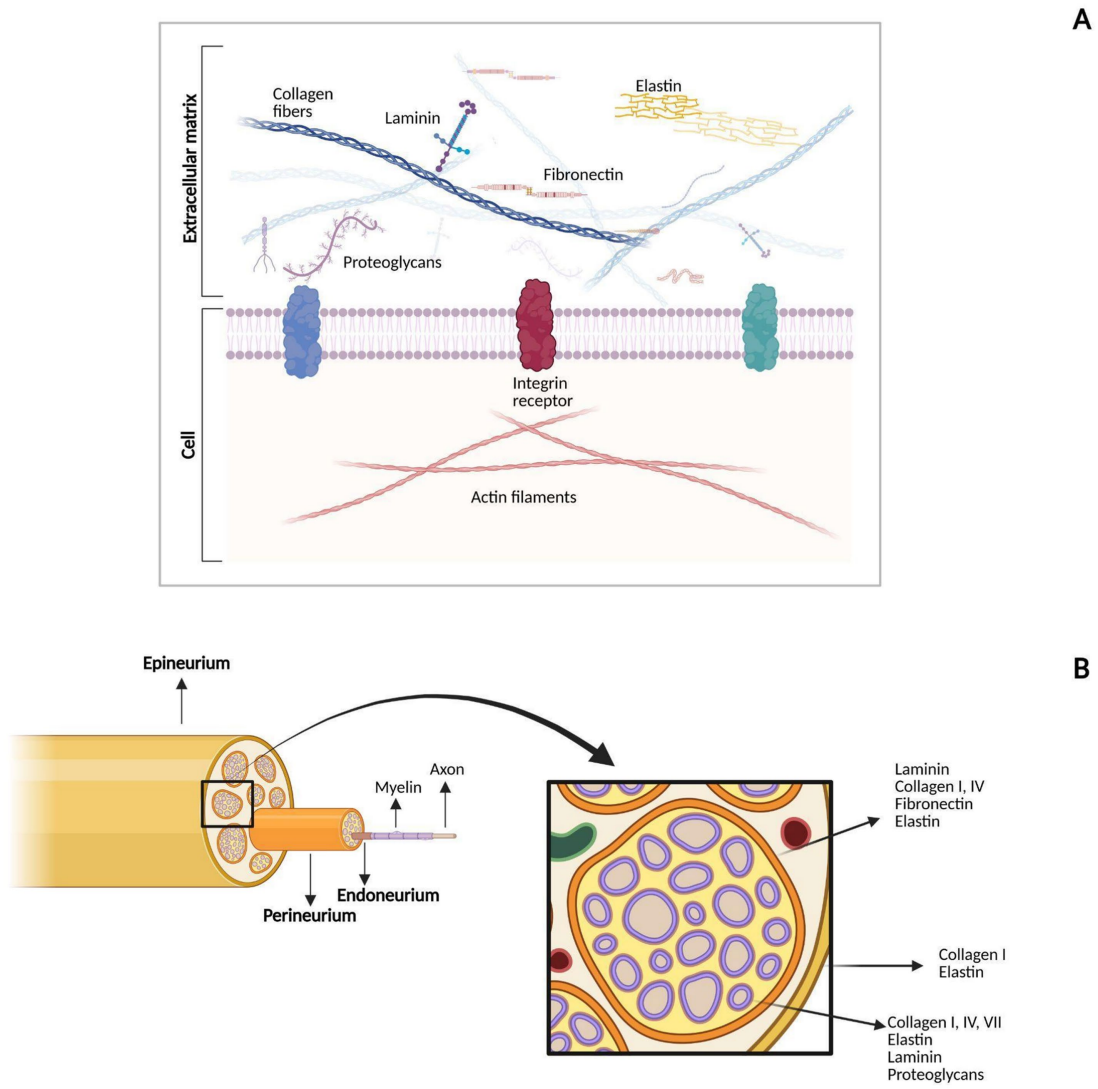
Image representing the composition and distribution of the ECM in peripheral nerves. **(A)** Shows the main components of the ECM, their distribution in the extracellular environment, and their interaction with cell surface receptors. **(B)** Shows how ECM components are distributed in the three different layers of connective tissue (epineurium, perineurium, and endoneurium). Created by BioRender.com.

The ECM of peripheral nerves is mainly composed of collagen, elastin, laminin, fibronectin and proteoglycans, which are differently distributed across the epineurium, perineurium and endoneurium (Figure 3). This section presents an overview of the main components of the ECM in peripheral nerves, highlighting their biochemical and structural features, and is supported by original illustrative images. Although these components have been described in detail in previous dedicated review articles (Gao et al., 2013; Gonzalez-Perez et al., 2013; Reichardt and Tomaselli, 1991), we present them here to provide essential background and context for the current review, particularly to support the discussion in the following sections on their roles in peripheral nerve repair.

### 2.1 Collagen

**Figure 4:**
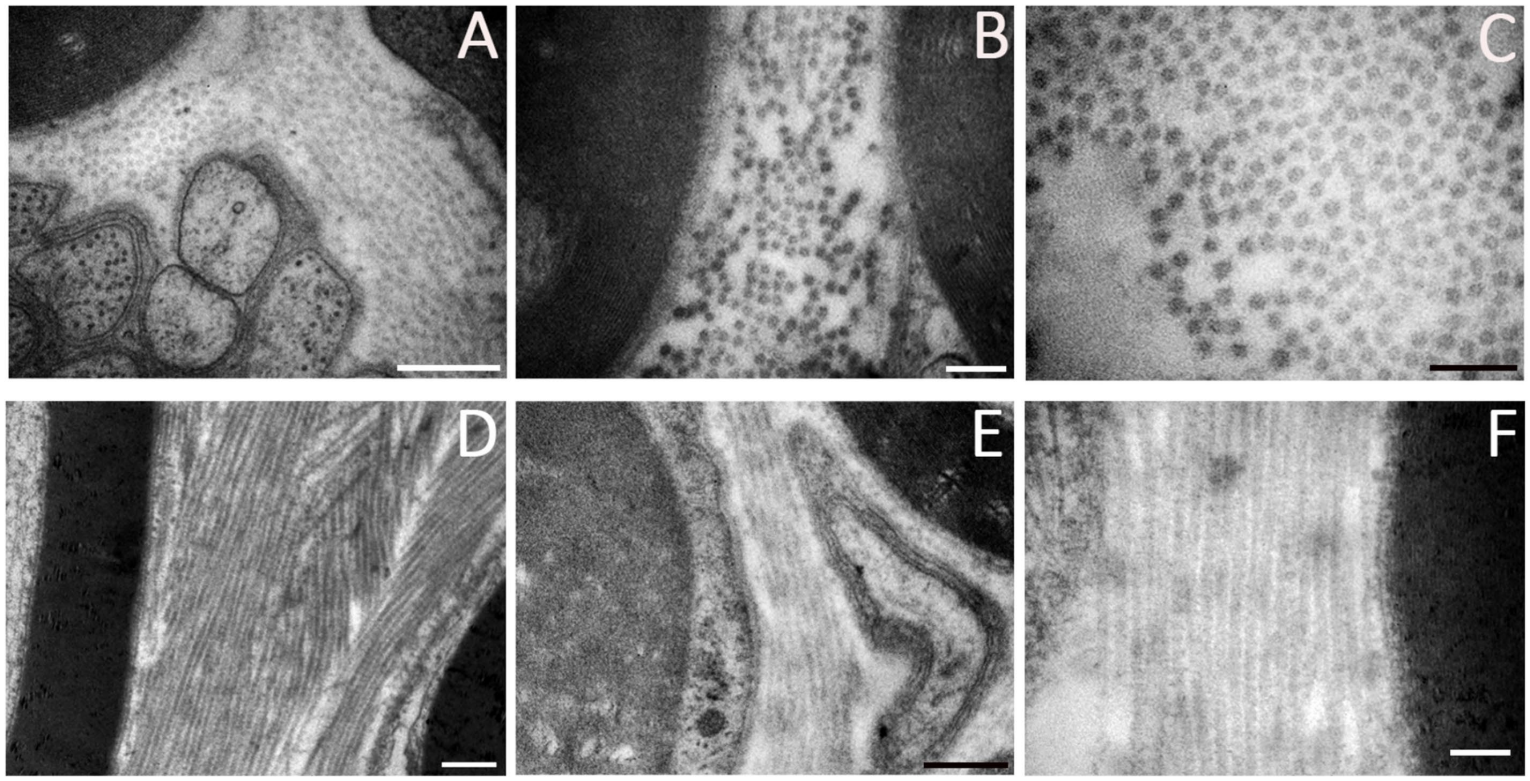
Ultrastructural details of ECM components in a healthy peripheral nerve. **(A)** Collagen fibrils among myelin sheaths and unmyelinated nerve fibers. **(B)** Collagen fibrils between myelinated nerve fibers. **(C)** Higher magnification showing transversal disposition of collagen. **(D–F)** Longitudinal collagen fibrils among nerve fibers. Scale bar: **(A-D-*F*)** = 0.5 μm, **(B-C-E)** = 0.2 μm.

Collagen (Figure 4) is a protein necessary for the ECM assembly. Collagen can be classified into several varieties, ranging from collagen type I to XXIX. These proteins are found in all three layers of connective tissues of the peripheral nerves as a component of the basal lamina enclosing SCs and perineurial cells, as well as lining the outer surface of endoneurial capillaries. In peripheral nerves, collagen is produced by fibroblasts, SCs, and perineurial cells; fibroblasts synthesize the majority of fibrillar interstitial collagen, while SCs and perineurial cells secrete the more extensively glycosylated and non-fibrillar type IV collagen (Jiang et al., 2024). Peripheral nerves are constituted primarily of type I collagen, accounting for around 90%. As recently demonstrated by Lu et al., after an injury, endoneurial fibroblasts produce type I collagen, which provides mechanical support for axonal growth and enhances SCs migration (Li A. et al., 2022; Lu et al., 2024). Collagen type IV is a key structural component of the basal lamina, released by SCs and perineurial cells, where it creates a covalently stabilized polymer network. The increase of collagen types I and IV following nerve injury promotes peripheral nerve regeneration (Gantus et al., 2006; Gao et al., 2013). This was further demonstrated by Lu et al., whose study reported restored motor function in rats after sciatic nerve repair using scaffolds filled with collagen type I (Lu et al., 2024). On the other hand, they demonstrated that type IV collagen can induce the proliferation of proinflammatory fibroblasts and scar formation, preventing proper regeneration (Lu et al., 2024). Type VI collagen is another type of collagen whose expression increases following nerve injury. Primarily produced by SCs, collagen type VI is considered a regulator of peripheral nerve regeneration. It promotes macrophage migration and polarization toward the M2 phenotype, regulates myelin thickness, and enhances axonal fasciculation by interacting with Neural Cell Adhesion Molecule 1 (NCAM1) receptors (Li A. et al., 2022; Lv et al., 2017). Indeed, Chen et al. (2015) demonstrated that the absence of collagen VI in Col6a1-null mice is associated with hypermyelination, an impairment of nerve conduction velocity and motor coordination, and a delayed response to acute pain stimuli. Furthermore, type V collagen promotes SC adhesion, spreading, and migration (Li A. et al., 2022), and types I, III, IV, and XVIII collagens support neoangiogenesis (Liu et al., 1997; Lohler et al., 1984; Poschl et al., 2004; Sertie et al., 2000).

### 2.2 Elastin

**Figure 5:**
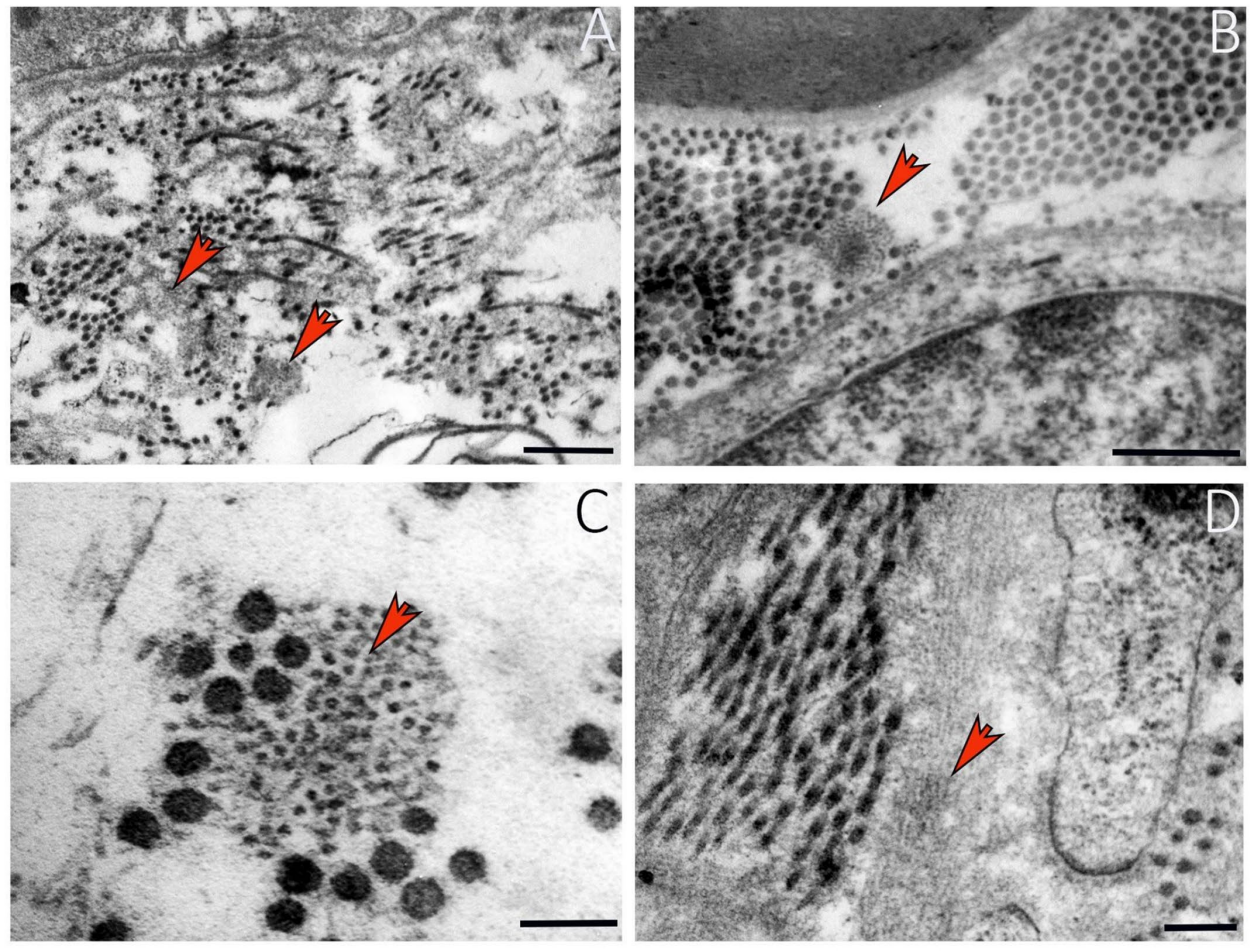
Ultrastructural details of elastin in a healthy peripheral nerve. **(A)** Elastic fibers among collagen fibrils (red arrows). **(B)** Elastin in endoneurium (red arrow). **(C)** Higher magnification showing organization of elastic fibers and collagen (red arrow). **(D)** Elastin ultrastructure in a longitudinal nerve section. Scale bar: **(A-B)** = 0.5 μm; **(C)** = 0.1 μm; **(D)** = 0.2 μm.

Elastin is an ECM protein present in all connective tissues, blood vessels, and nerves, providing them the ability to stretch when subjected to mechanical stress. It is produced by a variety of cells, including fibroblasts and endothelial cells (Yi et al., 2017). In peripheral nerves, the elastic fibers (Figure 5) are located consistently in the epineurium, in the perineurium and are widely distributed between the axons through the endoneurium (Tassler et al., 1994). However, the overall content of elastin is less compared to collagen, suggesting that the elastic properties of peripheral nerves may be due primarily to collagen (Tassler et al., 1994). An important property of elastin, according to Jiang et al. (2024) is its ability to enhance the elasticity and flexibility of myelin sheets after peripheral nerve regeneration.

### 2.3 Laminin

**Figure 6:**
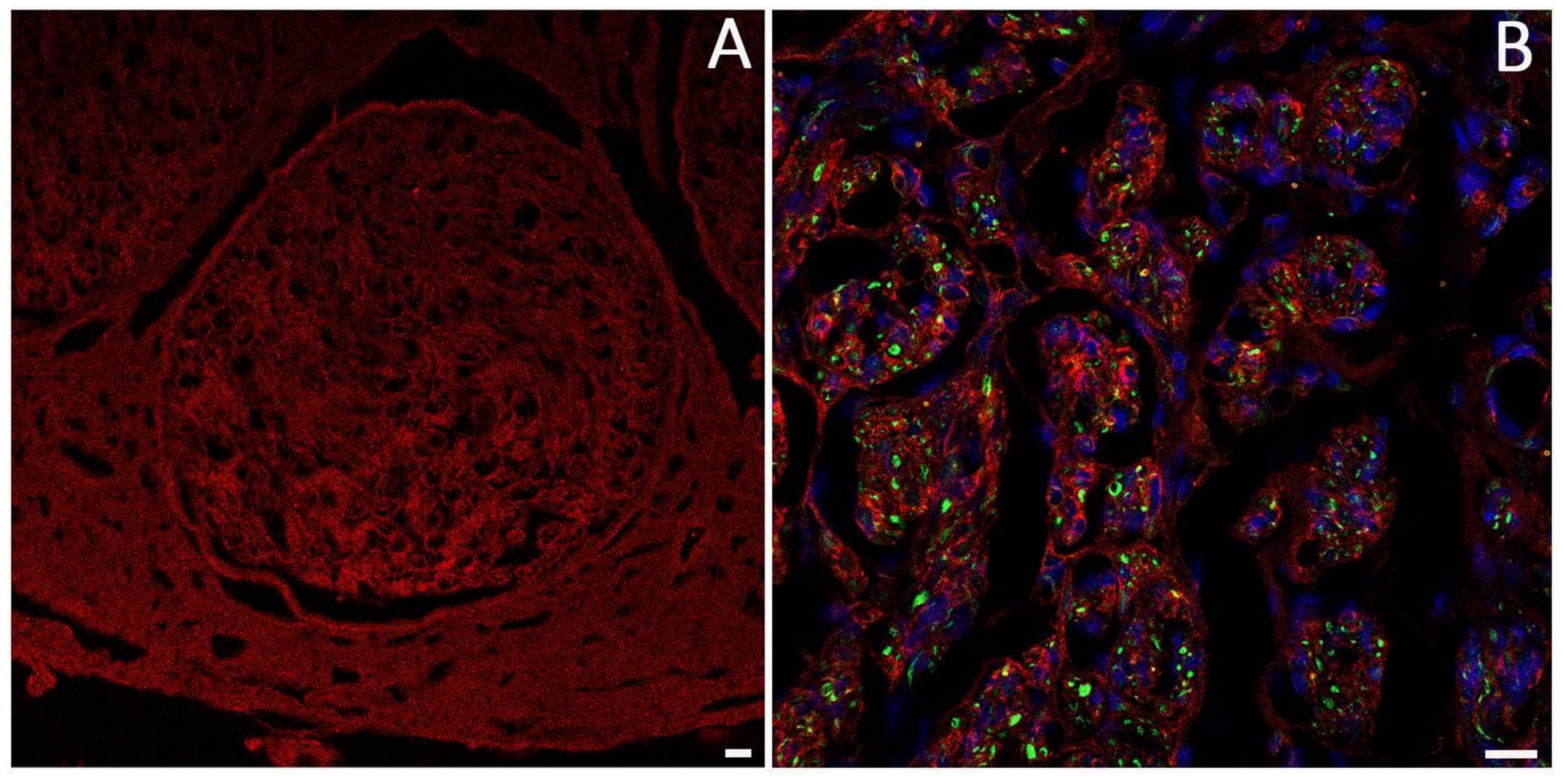
Immunofluorescence analysis using anti-laminin antibody (red) shows the organization and localization of laminin in the nerve. **(A)** Nerve fascicle; **(B)** laminin organization in a regenerating nerve, axons are labeled with anti-Pan neurofilament (green), and cell nuclei are stained with DAPI (blue). Scale bar = 20 μm.

Laminin is a large glycoprotein primarily located in the endoneurium and perineurium of peripheral nerves (Figure 6), where SCs secrete and store it within the ECM. Laminin is distributed along a continuous band, forming the basal lamina and interacting with other ECM proteins. It plays a key role in peripheral nerve development and regeneration by supporting SC migration (Lu et al., 2024; Yu et al., 2007). Its degradation after injury is considered a limiting factor in axonal regeneration (Klimovich et al., 2021).

Among various types of laminin, laminin-2 (merosin) is the most common isoform found in the basal lamina of SCs, with a composition of α2β1γ1 chains (Chernousov and Carey, 2000). Laminin-2 is critical for the establishment and maintenance of SC-axon interactions in both physiological and regenerating conditions (Uziyel et al., 2000). This evidence is supported by the observation that in *dy/dy* mice, an animal model of human merosin-deficient congenital muscular dystrophy, the reduction of mRNA level for the α2 laminin chain is accompanied by a decrease in the number of myelinated axons. This is due to the failure of the SC to properly ensheath axons, a crucial step for effective nerve regeneration (Uziyel et al., 2000). Similarly, the absence of the laminin α4 chain hinders the interaction between SCs and axons during peripheral nerve regeneration (Previtali et al., 2008). Furthermore, it has been reported that laminin α2-chain is overexpressed during the early stages of nerve regeneration, suggesting an important role in nerve repair (Gantus et al., 2006).

### 2.4 Fibronectin

Fibronectin is a glycoprotein found in the ECM of numerous tissues. In peripheral nerves, fibronectin produced by SC is distributed across the cell surface, with the majority localized in the perineurium. Fibronectins have binding sites for a range of ECM components and membrane receptors (integrins), playing key roles in the ECM construction and in a variety of cell activities such as adhesion, morphogenesis, and migration (Gonzalez-Perez et al., 2017). The most significant cell receptors for fibronectin are heterodimers of the integrin β1 type. In developing nerves, fibronectin and α5β1 integrin are highly expressed, with α5β1 integrin found in axons and non-neuronal cells (Gardiner et al., 2007). In intact adult peripheral nerves, the synthesis of these proteins is markedly reduced; however, their expression is significantly upregulated following injury, indicating a pivotal role for fibronectin in the regenerative response of peripheral nerves.

Following nerve injury, fibroblasts and endothelial cells locally synthesize fibronectin at the lesion site under the stimulation of TGF-ꞵ1 (Lefcort et al., 1992). The upregulation of fibronectin mediates successful nerve regeneration in two ways: (i) promoting the proliferation and migration of SC to the injury site, which is essential since axons fail to regenerate in their absence (Hall, 1986); (ii) acting as a substratum for the regenerated axons and supporting vascularization (George et al., 1993). Indeed, Gardiner et al. (2007) demonstrated that after a nerve injury, neurons interact with fibronectin, in particular with the 50 K fragment, which is a ligand of α5β1 integrin and produces neurites. More recently it has been demonstrated that fibronectin can boost SC proliferation and direct migration *in vitro*, potentially improving peripheral nerve lesion repair outcomes (Torres-Mejia et al., 2020).

### 2.5 Proteoglycans

Proteoglycans are complex glycosylated proteins present in nearly all ECM of connective tissues. The glycoconjugate component of proteoglycans is composed of a family of polysaccharides called glycosaminoglycans (GAGs) covalently attached to the protein component (Yanagishita, 1993). GAGs have a wide range of functions in biological behaviors, including cell adhesion, differentiation, proliferation, and ECM formation. Under both healthy and pathological conditions, these functions primarily depend on the specific structure of the GAG chains. Based on the glycidyl residues, the type of bond between them, and the number and position of the sulfuric groups, four main types of GAGs can be distinguished: hyaluronic acid, chondroitin sulfate, dermatan sulfate, heparan sulfate, or heparin. GAGs interact with other biological molecules, such as matrix proteins, growth factors, chemokines, and proteases. Indeed, GAG interaction with collagen improves the mechanical stability of the ECM. GAGs can work as “storage” components, hiding biological molecules from their inhibitory factors or proteolytic enzymes, allowing the ECM to create chemotactic gradients. The peripheral nervous system (PNS)’s response to the lesion is largely dependent on the balance of the various GAGs in the microenvironment surrounding the lesion. The presence of chondroitin sulfate proteoglycans (CSPG) generally corresponds to a hostile environment for nerve recovery, acting as inhibitors of axonal regeneration (Snow et al., 2001; Yang and Schnaar, 2008) however, there are some exceptions. According to Lin et al., chondroitin 6-sulphated (CS-6) GAGs make the ECM more permissive for axonal regeneration. Chondroitin 6-sulfotransferase-1 (C6ST-1), the enzyme that catalyzes the formation of CS-6 GAGs, has been shown to be up-regulated following peripheral nerve injury. In C6ST-1 knockout mice, which lack the ability to produce CS-6 GAGs via this pathway, peripheral nerve regeneration still occurs at levels comparable to wild-type mice. This is attributed to a compensatory mechanism involving the upregulation of another enzyme, chondroitin 6-sulfotransferase-2 (C6ST-2), which also contributes to the synthesis of CS-6 GAGs. These findings suggest that CS-6GAGs are beneficial for peripheral nerve regeneration and that the body can adapt to maintain their levels through alternative enzymatic pathways when necessary (Lin et al., 2011).

The upregulation of CSPG in the PNS after lesion is correlated with the failure of axonal regeneration (Gardiner, 2011). Although the exact mechanisms underlying this effect remain unclear, several hypotheses have been proposed. One possibility is that CSPGs disrupt the function of key organelles within the growth cone, such as mitochondria and the endoplasmic reticulum, thereby hindering axonal growth (Sainath et al., 2017); another suggested mechanism is that CSPGs interfere with cell-matrix interactions by masking ECM binding sites or by impairing integrin-mediated signaling pathways (Gardiner, 2011). Clear information about the distribution of CSPGs in the PNS is currently lacking. According to Muir et al., while CS-6 proteoglycan is associated with laminin in the SC basal lamina, chondroitin-4 sulphated (CS-4) GAGs are found in the endoneurium with an unclear function (Muir, 2010). Other information regarding the presence of CSPGs in the PNS concerns versican. Versican is a proteoglycan that occurs in 5 isoforms, ranging from V0 to V4. V1 versican is present in the perineurium and along the endoneurium’s basal lamina, where it prevents axons from growing out of the fascial boundary. Versican acts as an inhibitory barrier at the injury site, forcing growing axons along the designed paths (Liu et al., 2023). Liu et al. (2023) showed that axonal regeneration is effectively promoted by silencing the Chsy1 gene, which codes for the enzyme required for versican assembly in the PNS.

Among sulfate GAGs, there is perlecan, a large proteoglycan with a core protein. It is present in basement membranes, including the SC basal lamina. It has been reported that the core protein of perlecan binds fibronectin and is present in the fibrillar matrix deposited by SCs. Perlecan can also bind other ECM proteins, including laminin and various collagens, via its covalently bonded heparan sulfate chains (Chernousov and Carey, 2000). It regulates many cell signaling events through interactions with the FGF and the VEGF growth factor families to regulate vascularization (Melrose et al., 2021). However, details about the role of perlecan in peripheral nerve regeneration remain unknown. Agrin, a big proteoglycan located in the outermost layer of peripheral nerves’ myelin sheath, is another heparan sulfate GAG. It colocalizes with the *α*-dystroglycan protein, which serves as its receptor (Yamada et al., 1996). Although little is currently known about the function of agrin in the PNS, we do know that motor axons’ release of agrin causes acetylcholine receptors (AChRs) in postsynaptic membranes to aggregate, which is essential for the formation of neuromuscular junctions (Samuel et al., 2012; Zhang et al., 2006).

### 2.6 Other components: tenascin, matrilin and nidogen

Tenascin-C (TNC), tenascin-R (TNR), tenascin-X (TNX), and tenascin-W (TNW) are the four members of the glycoprotein tenascin family that have been found in vertebrate ECM. They are big oligomeric proteins that are mostly expressed during nervous system development. To date, knowledge regarding the potential role of these proteins in the context of peripheral nerves remains limited. It has been reported that TNC, which is expressed in a variety of tissues and cells, modulates cell adhesion and migration and is also involved in the regulation of neurite outgrowth and guidance (Zhang Z. et al., 2016; Zhang B. G. et al., 2016). Conversely, TNC has been described as an inhibitor of SC migration on fibronectin (Chernousov and Carey, 2000). Additionally, tenascin-X has been shown to be strongly expressed in peripheral nerves, particularly within the perineurium and endoneurium of sciatic nerves, where it is likely involved in blood vessel formation (Sakai et al., 2017).

Matrilin is an ECM protein family containing von Willebrand factor, a domain involved in the aggregation of protein complexes and in the development of ECM filamentous networks (Wagener et al., 2005). This family of proteins includes matrilin-2, which can bind to various ECM components, such as collagen, fibrillin, laminin, and fibronectin, contributing to the organization and structural integrity of the ECM structure (Malin et al., 2009). Similarly to tenascin, information about the role of matrilin in the regeneration of peripheral nerves is controversial. Although matrilin-2 knockout mice do not exhibit any obvious phenotypic abnormalities (Mates et al., 2004), Malin et al. (2009) demonstrated that matrilin-2 promotes SC migration and axonal outgrowth of dorsal root ganglia (DRG) neurons, suggesting a potential role in the peripheral nerve regeneration process of adult animals. This hypothesis was recently confirmed by Putman et al., who reported that matrilin-2 is overexpressed following nerve injury, regulating not only axonal growth but also modulating the immune response with both pro-repair and pro-inflammatory effects (Putman et al., 2025).

Nidogen is a ubiquitous protein of basement membranes. Its amino acid repeats allow it to bind to laminin and type IV collagen, enabling the assembly of the basement membrane (Chung and Durkin, 1990). The nidogen-SCs interaction promotes neurite growth from adult sensory neurons by inducing the expression of adhesion molecules by SCs and by stimulating SC proliferation and migration (Lee et al., 2009). Additionally, they showed that axons seem thinner and shorter when nidogen activity is hindered, highlighting the involvement of the protein in the axonal elongation phase (Lee et al., 2009).

## 3 Bioactive factors

Beyond providing structural support, the ECM also serves as a reservoir of soluble growth factors, thereby regulating their distribution, activation, and presentation to cells. The ECM of peripheral nerves is composed of a wide array of bioactive molecules secreted by SCs, macrophages, fibroblasts, neurons, and endothelial cells, all of which contribute to the regenerative process. Among the most relevant factors are nerve growth factor (NGF), fibroblast growth factor (FGF), vascular endothelial growth factor (VEGF), platelet-derived growth factor (PDGF), insulin-like growth factor 1 (IGF-1), transforming growth factor beta (TGF-*β*), and hepatocyte growth factor (HGF). Each of these molecules exerts specific and often synergistic actions, activating signaling pathways that sustain cell survival, proliferation, angiogenesis and axonal regrowth, ultimately enabling functional recovery after injury.

Among the different growth factors stored in the ECM, NGF was the first to be identified as a crucial regulator of nerve regeneration. It plays a fundamental role in neuronal growth, maintenance, and survival, while facilitating myelin debris clearance, thus creating a favorable regenerative environment (Li et al., 2020). NGF exerts its functions through interaction with two types of receptors: the tyrosine receptor kinase A (TrkA) and the p75 receptor (Chao, 2003). It is primarily produced by SCs (Gulati, 1998), who are prompted to release it by macrophage infiltration, which initiates debris removal (Gulati, 1998). There is also evidence that endothelial cells contribute to NGF production: according to Gibran and colleagues, substance P released from injured sensory nerve fibers stimulates endothelial cells to synthesize and release NGF, thus contributing to the regenerative process (Gibran et al., 2003).

In addition to NGF, another factor with a multifaceted role is FGF-2 (basic FGF or bFGF). It stimulates fibroblast proliferation, angiogenesis, SC and endothelial migration, while also enhances myelin clearance through autophagy activation. Its activity is stabilized and potentiated by binding to heparan sulfate proteoglycans (Schultz and Wysocki, 2009; Schlessinger et al., 2000), which facilitate its interaction with its receptors, particularly in the early post-injury phase (Jiang et al., 2021). Furthermore, an *in vivo* study has shown that FGF-2 overexpression in SCs via lentiviral transfection accelerates muscular reinnervation and stimulates neuronal regeneration (Allodi et al., 2014). While FGF-2 mainly sustains early responses, VEGF integrates vascular and neuronal functions, underscoring the interplay between angiogenesis and axonal regrowth. Best known for its angiogenic properties, it also exerts neurotrophic and neuroprotective effects (Jin et al., 2002) and low levels of it are associated with neurodegenerative disorders (Storkebaum et al., 2004). Indeed, it stimulates axonal growth, and its presence in the early phases of nerve regeneration is necessary for effective lesion repair (Huang et al., 2024). VEGFA is secreted by various cells present in the PNS, including SCs, macrophages and endothelial cells. Through VEGFR-2 activation and ERK signaling, it enhances SC proliferation while simultaneously driving neovascularization (Wu et al., 2021). Importantly, these new vessels act not only as nutrient suppliers but also as structural scaffolds that guide axonal regrowth, allowing regenerative cells to migrate across the nerve gap (Cattin et al., 2015).

IGF-1 is another central regulator of nerve regeneration, and its effects are primarily mediated by the signaling pathways involving PI3K-Akt and MAP kinase (Slavin et al., 2021; Rabinovsky, 2004). The first evidence of IGF-1 released by SCs following injury came from the study by Hansson and colleagues in 1986 (Hansson et al., 1986). This was followed by numerous studies demonstrating the important role of IGF-1 during nerve repair. IGF-1 regulates a number of processes, such as axonal sprouting, motor neuron elongation, and SC activity (Ishii et al., 1994; Rabinovsky, 2004). It is secreted not only by SCs but also by capillaries, skeletal muscle, and monocytes (Rabinovsky, 2004). Regarding its critical role during nerve regeneration, Gorio et al. (2001) provided evidence that exposure to high levels of IGF-1 induces nerve sprouting in adult skeletal muscle. Furthermore, IGF-1 serves as a key anti-apoptotic factor, conferring protection to SCs and neurons against apoptosis while concurrently promoting nerve sprouting (Delaney et al., 2001).

Together with IGF-1, PDGF provides synergistic signals that reinforce cell proliferation and survival, thereby ensuring a stable regenerative environment. It is a key regulator of cell growth, mainly implicated in the development of kidney cells and neural crest–derived cells (Oya et al., 2002). The specific member of the PDGF family that is part of the ECM of peripheral nerves is PDGF-B, whose receptor is PDGFR-*β* (Heldin and Westermark, 1989; Oya et al., 2002). During axonal regeneration, PDGF is synthesized by SCs, where it acts both as an autocrine mitogen and as an anti-apoptotic factor, working in synergy with IGF-1 (Sowa et al., 2019). In addition, similar to VEGF, PDGF not only exerts neuroprotective functions but also contributes to blood vessel formation by promoting pericyte differentiation (Basu et al., 2022).

TGF-*β* is a well-characterized factor known to play key roles in physiological processes such as cell proliferation, differentiation, tissue repair, and immune regulation. In peripheral nerve regeneration, it is secreted by resident macrophages (Sulaiman and Gordon, 2002), SCs (Li et al., 2014), and fibroblasts (Clements et al., 2017). The study of Sulaiman and Gordon highlights the specific contribution of TGF-β1, showing that it modulates the SC response to injury by promoting their proliferation and driving the shift from a myelinating to a proliferative activated phenotype (Sulaiman and Gordon, 2002). Moreover, TGF-β1 induces the redistribution of β1 integrins within growth cone filopodia (Gardiner, 2011), thereby facilitating interactions with other essential ECM components and supporting the success of the regenerative process.

Finally, HGF, predominantly secreted by fibroblasts, is a neurotrophic and neuroprotective factor (Ko et al., 2018) involved in the survival and proliferation of various cell types, acting through the activation of the c-met receptor (Birchmeier et al., 2003). HGF/c-met interaction in peripheral nerves induces neurite growth and proper innervation of target tissues (Maina et al., 1997) and acts on SCs by positively regulating their proliferation and migration, thus promoting the regenerative process (Ko et al., 2018). According to Lee et al., HGF is one of the first factors released after injury, and its interaction with axonal and SC c-met receptors is essential for the regenerative process, increasing the expression of genes associated with calcium influx at the injury site, an event crucial for initiating the regenerative cascade (Lee and Wolfe, 2000).

By coordinating cellular behavior, angiogenesis and axonal elongation, the ECM reservoir of bioactive molecules ensures an efficient repair process, ultimately enabling functional recovery after nerve injury.

## 4 Extracellular matrix receptors and signaling

**Figure 7:**
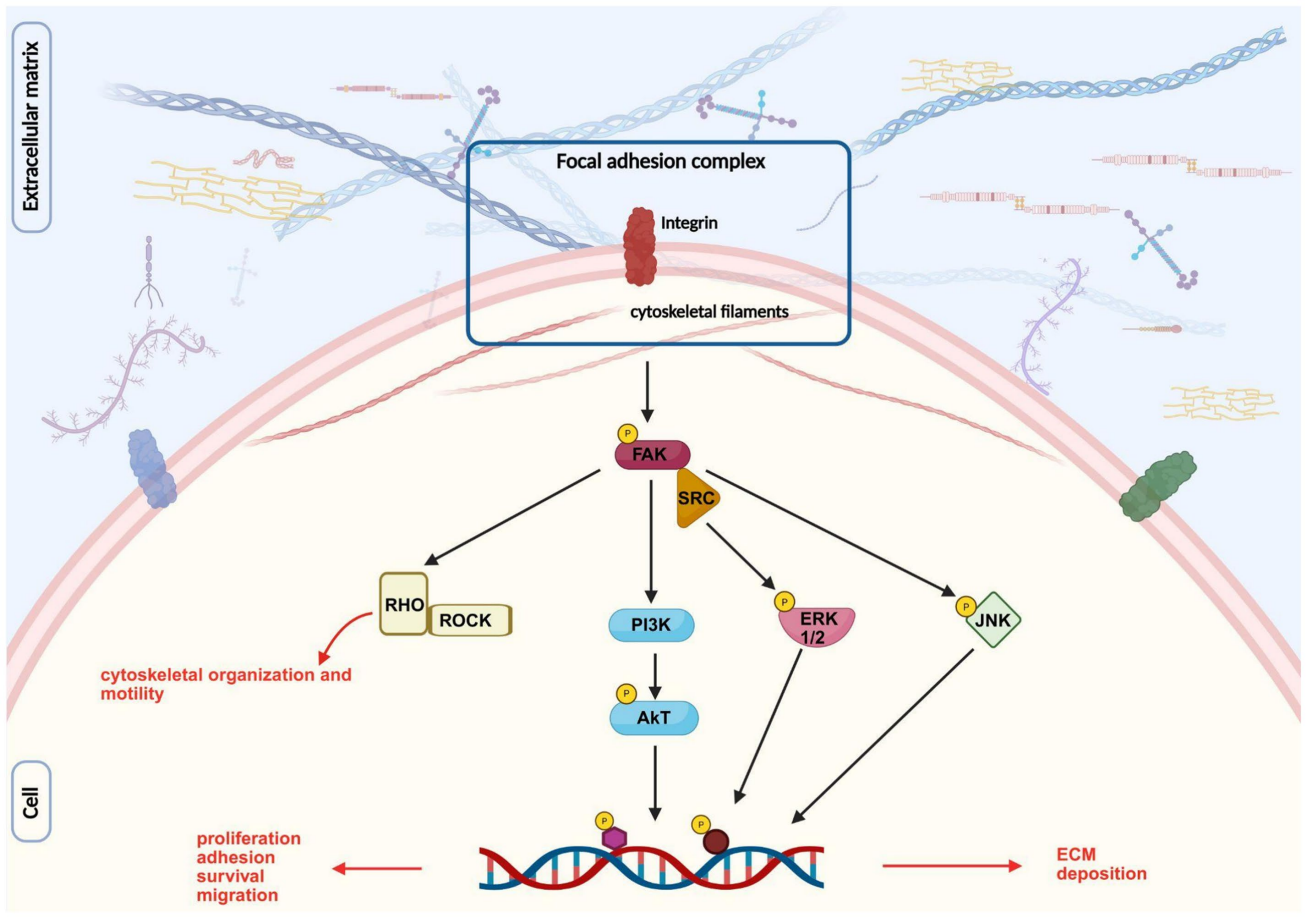
Image representing integrin signaling pathway after its interaction with ECM components. Inspired by Hastings et al. (2019), created with BioRender.com.

The function of the ECM is primarily mediated through its interactions with integrins, the most abundant receptors present on both neural and non-neural cells of peripheral nerves (Reichardt and Tomaselli, 1991). Integrins are transmembrane heterodimer receptors composed of two non-covalent subunits, *α* and *β*; the combination of dimers regulates the specificity for their ligands, resulting in binding ECM molecules with different affinities (Xu et al., 2024). Once bound to the ECM ligands, a process known as “inside-out” signaling, integrins build up the focal adhesion complex, comprising several cytoskeletal proteins (talin, kindlin, vinculin, paxillin, and actin). The formation of such complexes leads to the activation of focal adhesion kinase (FAK), proto-oncogene non-receptor tyrosine kinase (SRC), and integrin-linked kinase, which in turn activate canonical signaling pathways involving ERK, JNK, AKT, RHO/ROCK or small GTPases (Xu et al., 2024). This activation causes the transcription of genes involved in proliferation, migration, and axonal elongation (Figure 7).

Moreover, FAK and SRC have been shown to be associated with the formation of growth cone point contacts and the regulation of guidance cues (Xu et al., 2024).

In addition to integrins, other ECM receptors located on both neural and non-neural cells in peripheral nerves are proteoglycans, dystroglycans, and cell adhesion molecules (CAMs).

Cell surface proteoglycans containing heparan sulfate have been proposed as key mediators of ECM interactions, primarily functioning as co-receptors for ECM proteins to support the full spectrum of SC-ECM interactions (Chernousov and Carey, 2000). Several types of proteoglycans have been identified on SCs. Glypican-1, a lipid-anchored proteoglycan, is located on the outer surface of the SC membrane, where it interfaces with the laminin-rich basal lamina. Glypican-1 is the primary receptor for α4(V) collagen on the surface of SCs. The specific suppression of expression of α4(V) collagen or glypican-1 significantly reduces the amount of myelin that is formed in SC-DRG neuron cocultures, indicating that the glypican-1-collagen V interaction stimulates myelination (Chernousov and Carey, 2000).

The transmembrane proteoglycan syndecan-3, also known as N-syndecan, has a short cytoplasmic tail that facilitates interactions with signaling and structural proteins within SC (Chernousov and Carey, 2000).

Dystroglycans are receptors of ECM proteins in many mammalian tissues, including peripheral nerves, and are composed of two protein subunits: α-dystroglycan and β-dystroglycan. The α-subunit is located on the cell’s outer surface and forms a strong noncovalent association with the transmembrane β-subunit. The intracellular domain of the receptor interacts with cytosolic proteins, most notably those of the dystrophin family (Nickolls and Bonnemann, 2018). Together, the α and the β subunits and dystrophin represent the core functional unit of the dystrophin-glycoprotein complex, physically linking laminin-2 and cytoskeletal elements (Chernousov and Carey, 2000). Selective deletion of SC dystroglycan causes abnormal myelin sheath folding (Saito et al., 2003). Despite these findings, the potential role of these ECM receptors in peripheral nerve regeneration is not well known, requiring further investigation.

Another type of ECM receptor is cell adhesion molecules (CAM). CAMs include neural cell adhesion molecule (N-CAM), neural glial cell adhesion molecule (NG-CAM), myelin-associated glycoprotein (MAG), and peripheral myelin protein (PMP), among others. ECM and CAMs, in the early stages of nerve regeneration, regulate the axonal elongation and growth rate, promote cell adhesion, and maintain the stability of growth cone advancement (Jiang et al., 2024).

## 5 The role of the extracellular matrix during nerve regeneration

The success of nerve regeneration relies on the coordinated and dynamic interactions within the microenvironment of a regenerating nerve. ECM proteins play a crucial role by binding to specific cell surface receptors on SCs, axons, fibroblasts, and endothelial cells, thereby supporting the regenerative process (Reichardt and Tomaselli, 1991). If the ECM remodelling is disrupted during nerve regeneration, the nerve’s ability to regenerate may be compromised. For this reason, it is important to understand the biological mechanisms underlying the interaction of the ECM with cells involved in nerve regeneration.

### 5.1 Interaction of ECM proteins with Schwann cells

SCs are able to release ECM molecules, forming their basal lamina, and to interact with them during peripheral nerve regeneration (Jiang et al., 2024). The SC membrane contains integrin-specific αβ subunits that bind collagens (α1β1, α2β1), laminin (α2β1, α6β1, α6β4), and fibronectin (α5β1, αvβ3) as primary ligands (Chernousov and Carey, 2000). In addition to integrins, SCs present other membrane receptors, such as membrane proteoglycans, which facilitate interaction with ECM components and regulate SC behavior. The expression of these cell surface receptors can be regulated by various events. An *in vitro* study demonstrated that the physical contact between the NG108-15 neuronal cell line and SCs can alter the expression of SC surface receptors, triggering the release of neurotrophic factors through the activation of the MEK signaling pathway. Additionally, SCs upregulate the expression of integrins when co-cultured with DRG neurons (Armstrong et al., 2007). The interaction between SCs and ECM molecules enhanced neurite outgrowth by stimulating the release of NGF from SCs. However, neurite elongation did not occur only in the presence of diffusible factors (NGF, BDNF, CNTF), suggesting that direct SC-ECM interactions are essential for this process (Armstrong et al., 2007). It has been proposed that laminin binding to its receptor on SC activates the transcription factor NF-kB, leading to increased expression of CAMs, which in turn supports neurite growth (Armstrong et al., 2008). Additionally, the interaction between laminin and β1 integrins on SCs during peripheral nerve regeneration is thought to contribute to the formation of the myelin sheath (Chen and Strickland, 2003).

### 5.2 Interaction of ECM proteins with axons

ECM molecules not only provide structural support as a tissue scaffold but also actively interact with regrowing axons. Following nerve damage, growth cones interact with ECM components through integrin receptors, forming specialized adhesion structures known as “point contacts.” These point contacts link ECM to the actin cytoskeleton through talin, paxillin, and vinculin proteins, thereby modulating the retrograde flow of filamentous actin (F-actin) driven by myosin II. This interaction triggers the RhoA-ROCK signaling pathway, promoting F-actin contractile forces of migrating cells and stimulating growth cone advancement (Nichol et al., 2016).

Laminin and collagen are known to be the most effective ECM promoters for neurite extension. Proper interaction between the SC basal lamina and regrowing axons is essential for effective axonal regeneration, and the delay of this interaction may cause axonal degeneration (Namgung, 2014). Furthermore, following peripheral nerve damage, the interaction between SCs β1 integrin and laminin facilitates neurite outgrowth of DRG neurons co-cultured with SCs, enhancing microtubule assembly and stabilization (Chang et al., 2012). Among the various types of collagen in the ECM, type VI collagen has been identified as an important signal that promotes axonal fasciculation and enhances the distribution of NCAM1 receptors in the axolemma, thereby improving nerve regeneration after injury (Su et al., 2021). However, the type VI collagen-NCAM1 interactions in the nervous system, along with the regulatory mechanisms of type VI collagen that are linked to axonal fasciculation, are still unknown.

### 5.3 Interaction of ECM proteins with fibroblasts

Fibroblasts are known to produce ECM molecules to maintain the structural integrity of tissues; their capacity to communicate with other cells makes them a central component during tissue repair (Sorrell and Caplan, 2009; Zhang Z. et al., 2016; Zhang B. G. et al., 2016). For this reason, growing attention has been directed toward understanding the role of fibroblasts during nerve regeneration with the goal of uncovering new insights and therapeutic strategies in this area. Following peripheral nerve injury, a large number of fibroblasts accumulate at the injury site and secrete new ECM components to regulate the regenerative microenvironment and promote axonal regeneration (Qian et al., 2018; Zhang Z. et al., 2016). Indeed, the distribution of fibroblasts changes during the different phases of nerve regeneration. Fibroblasts concentrate at the proximal stump as soon as the injury occurs, and within 4 days, they move from the proximal to the distal stump. Fourteen days following the lesion, they cluster in the nerve’s epineurium and perineurium (Qian et al., 2018; Zhang Z. et al., 2016).

In these stages, fibroblasts release pro-regenerative factors that control neurite growth, such as neuregulin, and produce ECM molecules, such as laminin and type I and IV collagen, to restore tissue structure (Li X. et al., 2022; Obremski et al., 1993). Additionally, fibroblast-derived factors can modulate the production of matrix components by SCs, thereby playing a comprehensive role throughout the different stages of nerve regeneration (Dreesmann et al., 2009; van Neerven et al., 2013).

Fibroblasts directly interact with ECM proteins, engaging collagen and laminin via specific β1 integrin receptors (Liu et al., 2010; Zhang et al., 2006), including α2β1 (Elices and Hemler, 1989; Klein et al., 1991), thus orchestrating ECM structural organization. Furthermore, it has been demonstrated that fibroblasts of different phenotypes promote neurite growth in a subtype-specific manner, with sensory fibroblasts significantly stimulating sensory neuron neurites and motor fibroblasts significantly stimulating motor neurons (He et al., 2023). This difference is associated with the differential expression of integrin β1 (ITB1), with sensory neurons showing elevated receptor levels following injury, while motor neurons exhibit higher expression under normal conditions (He et al., 2023). However, the specific role of this differential receptor expression is still unknown and needs in-depth analysis.

### 5.4 Interaction of ECM proteins with endothelial cells

ECM provides biochemical and mechanical cues that regulate endothelial cell behavior, including migration, proliferation, and tube formation, to support newly formed blood vessels.

The formation of new blood vessels has a crucial role in nerve regeneration, not only by supporting cell survival but also by serving as a guide for SCs migration, which is necessary for proper axonal pathfinding (Fornasari et al., 2022).

The dynamic interaction between ECM components and endothelial cell surface receptors, such as integrins, influences the structural organization of blood vessels, contributing to the repair process (Saio et al., 2021). The interaction through endothelial cells and the main regulator of angiogenesis, VEGF, occurs through binding to the tyrosine kinase receptors VEGFR1 and VEGFR2 (Rhodes and Simons, 2007). Another component of the matrix that is important for blood vessel formation and stability is heparan sulfate proteoglycans. Jakobsson and colleagues have demonstrated that when sulfated, these proteoglycans can modulate the VEGFA gradient present in the extracellular environment and regulate VEGFR2-mediated signaling (Jakobsson et al., 2006). Collagen types I, III, IV, and XVIII bind endothelial cell surface integrins, such as α1β1 and α2β1 (Whelan and Senger, 2003), supporting the mechanical stability of blood vessels (Poschl et al., 2004; Sertie et al., 2000). Among other ECM components, laminin α4 is essential for the construction and maintenance of the capillary basement membrane (Thyboll et al., 2002). Indeed, mice with null alleles for Lama4, the gene encoding for the laminin α4 chain, showed weakening of capillary basement membranes and rupture of vascular walls (Thyboll et al., 2002). Fibronectin regulates endothelial cell proliferation, adhesion, and migration through its interaction with the integrin α5β1 receptor (George et al., 1993). It also contains two binding sites that modulate VEGF activity by promoting the association between α5β1 integrin and VEGFR2, thereby amplifying the signaling pathway that drives endothelial cell migration (Rhodes and Simons, 2007).

In conclusion, ECM coordinates angiogenesis by combining mechanical stability and biochemical cues, ensuring the proper formation and maintenance of vascular structures.

### 5.5 The role of ECM biophysical properties on PNS physiology and injury

Nerve ECM possesses distinct physical properties to support nerve structure and function, not only providing essential biochemical signals but also defining the mechanical microenvironment that regulates nerve development, homeostasis, injury response, and regeneration (Kong et al., 2022). Studies have demonstrated that during PNS development, ECM stiffness exhibits spatial heterogeneity and dynamic changes over time. Basic nerve ECM components such as collagen types I and IV and elastin significantly contribute to the physical properties of nerve tissue, whereas factors like cellular density or myelination exert comparatively less influence (Rosso and Guck, 2019). The biophysical characteristics of the ECM play a fundamental role in PNS physiology by influencing SC behavior, myelination, and nerve structure. SCs are mechanosensitive, detecting and responding to ECM stiffness, elasticity, and topography during nerve development, maturation, and regeneration. It is well established that as connective tissues and the basal lamina mature, the stiffness of the SC microenvironment progressively increases. This mechanical evolution is crucial for regulating cellular functions, correlating with enhanced myelin production, improved nerve fiber organization, and the functional maturation of SC phenotype. These biomechanical changes not only stabilize axon-SC interactions but also help establish a microenvironment that supports efficient nerve signal transmission and long-term peripheral nerve homeostasis (Belin et al., 2017; Manganas et al., 2022; Rosso and Guck, 2019; Urbanski et al., 2016). Following peripheral nerve injury, the ECM can undergo structural damage, altering its mechanical properties in a severity-dependent manner (Urbanski et al., 2016). Since biophysical properties of nerve ECM play a pivotal role in directing regeneration, the interplay between SCs and ECM during the repair process is critical. Indeed, ECM-derived cues guide SC behavior, while SCs are actively engaged in mechanotransduction, sensing and responding to microenvironmental signals that regulate their plasticity. Key features such as stiffness, elasticity, porosity, and topography modulate SC adhesion, proliferation, migration, and differentiation, thereby facilitating axonal regrowth and nerve repair (Wu et al., 2018). For this reason, alterations in ECM mechanics during the regeneration process, such as increased stiffness due to fibrotic scar formation, can hinder axonal growth and SC functions: indeed, it has been demonstrated that elevated collagen deposition and changes in basement membrane structure after peripheral nerve injury lead to increased local stiffness, which in turn activates mechanosensitive signalling promoting SC senescence. This latter condition is unfavorable to the repair mechanism, as it limits the proliferative and regenerative capacity of SCs, compromising nerve regeneration (Liang et al., 2024). For this reason, ECM stiffness is likely the most critical property influencing nerve repair. Supporting this, evidence indicates that the mechanosensitive ion channel Piezo1 is upregulated in SCs, triggering cellular senescence and impairing their ability to proliferate and migrate. This senescent phenotype contributes to excessive collagen deposition and persistent scarring, which negatively affect regeneration and functional recovery (Liang et al., 2024). To mitigate the adverse effects of increased ECM stiffness, recent biomaterials and tissue engineering strategies have focused on developing devices that closely replicate the native mechanical properties of the extracellular matrix. These biomimetic platforms help preserve a supportive microenvironment for SC viability, function, and regenerative potential, ultimately promoting nerve regeneration. Furthermore, through the integration of bioactive molecules and topographical cues, advanced scaffolds can modulate SC behavior and phenotype, thereby enhancing cellular alignment, migration, and differentiation to improve regeneration outcomes (Putman et al., 2025).

## 6 Methods for studying extracellular matrix during nerve regeneration process

Given the essential role of ECM in physiological, developmental, and reparative processes, studying this complex network in the context of nerve repair is crucial, not only for a better understanding of regenerative processes, but also to explore its potential for enhancing nerve repair strategies. ECM can be analysed using conventional and novel approaches, including histological staining, immunofluorescence, and advanced imaging techniques such as confocal and electron microscopy (Van De Vlekkert et al., 2020). Additionally, techniques such as mass spectrometry, along with biochemical and molecular analyses, offer valuable insights into the molecular composition, structural organization, and functional properties of the ECM.

### 6.1 Histological staining

The most basic technique to identify different components of the ECM is the employment of specific histological stainings. To detect collagen, the most abundant ECM macromolecule, commonly used stains include Trichrome and Picrosirius red stains. These stains are particularly valuable in studying collagen organization and remodelling during nerve regeneration, enabling the visualization of pathological changes such as scar tissue formation.

Particularly, Masson, Azan, and Mallory trichrome stains highlight collagen fibers in blue due to the presence of the acid dye aniline; variations of the dye stain collagen in light green.

Masson’s Trichrome staining employs a combination of three dyes: hematoxylin, which stains nuclei violet; acid fuchsin, which stains cytoplasm, muscle, and keratin red; and aniline blue or light green, which stains collagen fibers blue or green, depending on the dye variant used (Van De Vlekkert et al., 2020).

In the Azan method, two acid dyes are used: azocarmine and aniline blue. The combination of these two dyes results in a distinctive color contrast that facilitates the identification of tissue elements. This method highlights collagen fibers in intense blue (Miyauchi et al., 2025).

Mallory’s Trichrome stain combines aniline blue, acid fuchsin, and orange G to create vivid contrast among tissue components. In this method, collagen fibers stain blue, muscle fibers and cytoplasm appear red, erythrocytes are orange, and nuclei exhibit a reddish-purple hue (Rosario et al., 2022).

These stains enable clear visualization of collagen distribution and density, providing valuable insights into fibrotic processes, tissue remodeling, scar tissue formation, and structural changes within the ECM, which are essential for assessing tissue integrity and regenerative outcomes in nerve repair.

Picrosirius red is an anionic dye binding to cationic collagen fibers, enhancing their natural birefringence under cross-polarized light (Junqueira et al., 1979; Montes and Junqueira, 1991). Several studies demonstrated that this staining is useful to observe collagen network abnormalities (Junqueira and Roscoe, 1985) and to identify collagen types according to their birefringence under polarized light: strong yellow-red birefringence is specific to collagen type I, and weak greenish birefringence is typical of collagen type III (Bayounis et al., 2011; Binnebosel et al., 2010; Cavallo et al., 2014; Lattouf et al., 2014). By analyzing the intensity and distribution of these colors, it is possible to quantitatively assess the relative amounts of different collagen types in a tissue. This makes Picrosirius red staining a valuable tool for studying fibrosis, wound healing, and various pathological conditions where collagen remodelling plays a crucial role (Lattouf et al., 2014).

Elastic fibers can be visualized using Verhoeff’s elastic van Gieson, also known as Verhoeff-Van Gieson (VVG), the most widely used method for detecting elastin. This staining technique combines two components: Verhoeff’s stain, which is specific for elastic fibers, and the van Gieson component, which highlights collagen, allowing for clear differentiation between tissue elements. In VVG-stained tissue, elastic fibers appear black, collagen appears red/orange, and other tissue elements appear yellow (Halabi and Mecham, 2018). Miller’s elastic stain, which is composed of three dyes (Victoria blue 4R, new fuchsin, and crystal violet), stains elastic fibers in black, collagen fibers in red, fibrin in yellow, and nuclei in green (Castro, 1989). These stains can be employed to assess the presence and remodelling of elastic fibers within the nerve during regeneration.

To visualize GAGs, Alcian blue staining is commonly used. This positively charged dye forms electrostatic bonds with negatively charged polyanions by binding to carboxyl or sulfate groups, resulting in stable Alcian Blue–GAG complexes. After forming the Alcian Blue-GAG complex, the amount of GAGs can also be quantified spectrophotometrically by measuring absorbance at 480 nm (de Jong et al., 1994). Alcian blue is useful for staining GAGs in the presence of other polyanions but has lower sensitivity compared to alternative methods such as dimethylmethylene blue (DMB). Like alcian blue, DMB is a cationic stain that binds to negatively charged GAGs, resulting in a color shift from blue to purple. This color change is directly proportional to the concentration of GAGs. The GAG amount is then measured using a spectrophotometer, typically at a wavelength of 520 nm (de Jong et al., 1994).

### 6.2 Immunofluorescence and confocal microscopy

Another typical technique that allows the visualization of ECM components is immunofluorescence (IF) through combinations of specific antibodies tagged with fluorophores. Consequently, its applications represent an advantageous approach to specifically identify and localize ECM elements, such as collagen, laminin (including different types like laminin-1, laminin-2, and laminin-5), and fibronectin, as well as collagen chains (type I, II, and IV collagen), exploiting the antigen–antibody binding.

In the context of nerve repair, IF is a valuable tool for assessing how the ECM interacts with regenerating tissue and undergoes remodeling during nerve regeneration. By using antibodies against both regeneration-related components, such as SCs and axons, and ECM molecules, IF enables detailed visualization of the dynamic interactions between these elements. Additionally, IF can be used to explore ECM-receptor interactions, offering deeper insight into how the ECM influences cell function and tissue repair during nerve regeneration.

### 6.3 Other advanced imaging techniques

Additional imaging techniques used to study the ECM are transmission electron microscopy (TEM) and scanning electron microscopy (SEM), which allow for the examination of ECM at the ultrastructural level. TEM offers high-resolution insights into fibrils and basement membranes, while SEM offers a 3D surface-level perspective (Starborg and Kadler, 2015), ideal for studying the interactions between cells and ECM. Both techniques are invaluable for understanding the dynamics of ECM remodelling during nerve repair.

Among biochemical and biomolecular techniques to study ECM composition during nerve regeneration, Western blot combines gel electrophoresis and immunoblotting to allow the identification of proteins of interest extracted from tissue, such as those against specific collagen chains, laminin, receptors, proteins involved in cell signaling, and other ECM proteins.

### 6.4 Biochemical and molecular analyses

To study the proteomic profile of ECM, the liquid chromatography–tandem mass spectrometry (LC–MS/MS) approach, followed by bioinformatic analysis, allows the identification and quantification of ECM proteins, providing findings about their roles and changes during nerve regeneration (Li L. et al., 2023). Since the ECM is abundant in glycoproteins and proteoglycans, high-performance liquid chromatography (HPLC) facilitates the quantification of carbohydrates present in the ECM. Using HPLC, carbohydrates like GAGs are enzymatically broken down, separated, and quantified. The enzymatic digestion is performed at different pH levels to differentiate between various types of GAGs (Grondahl et al., 2011). Another useful technique for quantifying GAGs is the lectin microarray technique, which offers a rapid, high-throughput method for analyzing glycans without the need to separate them from proteins. It utilizes lectins, proteins that specifically bind to carbohydrates, and involves immobilizing the lectins on a glass slide to detect glycans in biological samples. The lectin microarray is faster and requires fewer steps compared to traditional methods, such as mass spectrometry, and does not require glycan separation, thus preserving the natural structure of ECM glycoproteins like laminin and fibronectin (Gupta et al., 2010; Hirabayashi et al., 2015; Hu and Wong, 2009).

## 7 Nerve repair strategies that exploit the potential of the extracellular matrix to enhance regeneration

Severe nerve injuries often create a gap between the two ends of the damaged nerve that must be bridged to restore sensory and motor function. The most common treatment is an autograft; however, this method has limitations such as complications at the donor site and limited availability of suitable tissue (Rebowe et al., 2018). To overcome these challenges, researchers and clinicians are increasingly exploring alternatives, such as the use of bio-conduit.

A bio-conduit is a biologically compatible tube-like structure designed to guide and support the regrowth of nerve fibers across an injury site. Acting as a physical bridge, it helps align regenerating fibers and provides a protective environment. Bio-conduits can be made from natural materials (like collagen) and may also be enriched with cells or growth factors to promote faster and more effective nerve regeneration. For a comprehensive and up-to-date overview of the various biomaterials and scaffolds used in peripheral nerve repair, including their properties, mechanisms of action, and clinical applications, readers are referred to several review articles on this topic (Fornasari et al., 2020; Lam et al., 2025; Li et al., 2025; Wang et al., 2025).

**Figure 8:**
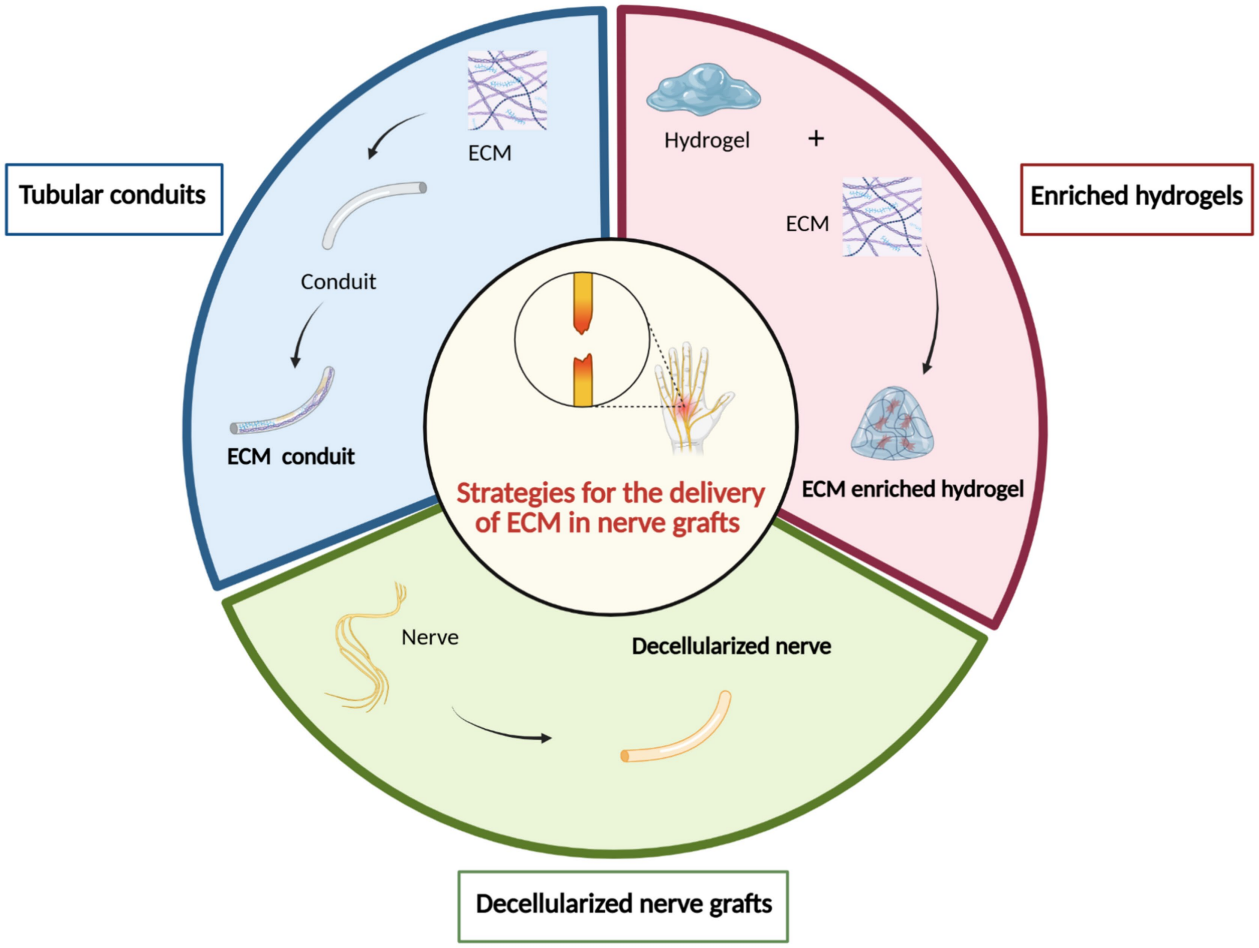
Strategies for the application of the ECM in nerve grafts. Three main approaches have been investigated to incorporate ECM components in peripheral nerve repair: (1) ECM tubular conduits to provide a bioactive guidance structure; (2) ECM-enriched hydrogels, used as intraluminal fillers within conduits to support axonal growth and regeneration; and (3) decellularized nerve grafts, which retain the native extracellular architecture and biochemical cues after removal of cellular components. These strategies aim to recreate a regenerative microenvironment that promotes functional nerve repair. Created by BioRender.com.

In particular, the development of bio-conduits that exploit the regenerative potential of the ECM has emerged as a highly successful strategy for promoting peripheral nerve repair (de Luca et al., 2014). Different strategies for the use of ECM components in nerve grafts have been described: (i) tubular conduits composed of ECM molecules, (ii) hydrogel enriched with ECM molecules as conduit fillers, and (iii) decellularized nerve graft implantation (Figure 8). These approaches will be discussed individually in the following paragraphs.

### 7.1 Tubular conduits composed of ECM molecules

Different ECM molecules have been used to develop conduits for the repair of severe peripheral nerve injuries (Fornasari et al., 2020). Biomaterial conduits composed of ECM-based molecules have shown the ability to guide cellular and tissue behavior during regeneration, thanks to their distinctive mechanical and bioactive properties. Among these, fibronectin has been demonstrated *in vitro* to improve the attachment of fibroblasts with the subsequent deposition of collagen (Branford et al., 2011), which is useful for the early phases of nerve regeneration.

In the early 1990s, fibronectin was first tested *in vivo* for repairing a 1 cm gap in the rat sciatic nerve (Whitworth et al., 1995). The study demonstrated that conduits made from unidirectionally aligned fibronectin membranes facilitated cellular alignment along the fibronectin pattern and significantly accelerated axonal regeneration.

About a decade later, Phillips et al. (2002) developed a fibronectin-based conduit using a viscous solution of concentrated plasma fibronectin derived from human plasma fractionation by-products. *In vivo* studies in rat sciatic nerve injury models confirmed fibronectin’s effectiveness as a guidance substrate for nerve fiber regrowth.

In more recent studies, fibronectin was primarily used in combination with other materials or as enrichment for conduits (Gonzalez-Perez et al., 2018; Mottaghitalab et al., 2013).

Collagen is the most extensively studied extracellular matrix component for nerve conduit fabrication. Accordingly, several FDA-approved collagen-based materials are available for use as nerve grafts, including NeuraGen^®^, NeuroFlex^™^, NeuroMatrix^™^, NeuraWrap^™^, and NeuroMend^™^ (Arslantunali et al., 2014). The most used, both in experimental models and in the clinical field, is NeuraGen^®^ Nerve Guide, a resorbable implant for repairing nerve injuries that provides a protective environment that allows controlled resorption, appropriate nutrient diffusion, and retention of growth factors.

Although collagen-based conduits have demonstrated to be effective, various molecules have been incorporated to further enhance their regenerative potential. For example, the layer-by-layer technique has been used to modify NeuraGen^®^ surfaces using heparin/collagen (HEP/COL) coating systems. This study demonstrates *in vitro* the reparative-enhancing potential of these extracellular mimetic coatings (Pinzon-Herrera et al., 2024).

The integration of natural polymers on the surface of substrates has gained significant importance in biomedical applications since it leverages the electrostatic charge interactions to create coatings that enhance surface properties. For example, coating a collagen scaffold with laminin and fibronectin has been shown to increase its effectiveness in promoting axonal regeneration, as demonstrated by enhanced functional recovery (Ding et al., 2011).

Other ECM components found in nerves have been rarely used alone to fabricate tubular conduits, mainly because of their limited mechanical strength. To overcome this limitation, they are frequently combined with other biomaterials. For instance, bilayered electrospun conduits have been developed, with an outer layer made of poly (L-lactic acid-co-*ε*-caprolactone) (PLA-PCL) and an inner layer of ECM derived from decellularized porcine sciatic nerve (Mao et al., 2023). In another study, laminin has been used to modify conduit surface, exploiting its positive influence on neurite outgrowth and growth cone chemotaxis (Adams et al., 2005).

Many studies proposing ECM molecules for the development of nerve conduits perform only *in vitro* analyses and do not evaluate the mechanical properties of the conduit. For instance, elastin-like polypeptides have been identified as a promising biomaterial due to their biocompatibility, supporting SC viability, adhesion, and proliferation. However, this study does not include *in vivo* evaluations (Hsueh et al., 2014).

White et al. (2015) also demonstrated only *in vitro* that small tropoelastin polymers can be used to form composite materials, such as silk–tropoelastin film blends, which effectively promote neurite growth (White et al., 2015).

### 7.2 Hydrogel enriched with ECM molecules

For the repair of nerve injuries with a large gap between the two nerve stumps, a tubular scaffold is essential to guide axonal regrowth and bridge the lesion. In fact, in peripheral nerve regeneration, the conduit not only enables the effective delivery of biochemical signals, but also provides the necessary physical guidance for regenerating axons to reach the distal stump directly, thereby minimizing the risk of misdirected sprouting or neuroma formation. However, the use of an empty conduit alone is often insufficient to support optimal regeneration. To enhance its regenerative potential, the lumen can be enriched with fillers such as hydrogel. Owing to their hydrophilic nature, three-dimensional architecture, and tunable topography, hydrogels support cell adhesion, proliferation, and migration (Jansen et al., 2018; Taisescu et al., 2025). When combined with ECM molecules in a composite scaffold, hydrogels can create an optimal biomimetic environment that promotes nerve fiber regrowth, while preserving structural integrity.

The most commonly used endogenous ECM components for luminal fillers in nerve repair include collagen, laminin, fibrin, and fibronectin (Chen et al., 2000; Pabari et al., 2011; Tusnim et al., 2024). In addition to these nerve-specific ECM molecules, a variety of other natural biopolymers, such as polysaccharides like chitosan, alginate, and hyaluronan, or fibrous protein gels such as gelatin, have been widely employed to develop filler for nerve scaffolds beyond simple tubular conduits (Huang et al., 2024). Furthermore, hydrogels derived from xenogeneic non-nerve-specific tissues, such as bone, liver, and small intestine (Kellaway et al., 2023), and umbilical cord-derived decellularized ECM (Fan et al., 2025) shown promising results in improving peripheral nerve regeneration. These findings support the continued exploration of increasingly sophisticated hydrogel-based scaffolds for advancing peripheral nerve repair strategies.

Polysaccharide-based hydrogels have garnered considerable attention in recent years due to their properties of maintaining their biocompatible nature and their applications in tissue engineering. The purification process, procedures to remove any impurities during the formulation of hydrogel, ensures that the hydrogels are nontoxic, thus posing minimal risk to the regenerating tissue. Moreover, polysaccharide-based hydrogels, due to their natural origin and resemblance to the ECM components, minimize the immune reactions (Tamo et al., 2024).

Purified ECM proteins, such as collagen and fibrin, have been extensively employed in peripheral nerve repair owing to their capacity to support axonal outgrowth throughout the regeneration process (Bell and Haycock, 2012; Gu et al., 2011; Wang and Cai, 2010). Their effectiveness likely stems from their physiological relevance: during the natural process of nerve regeneration, the formation of a fibrin-rich scaffold typically forms between the proximal and distal stumps within 1 week after injury, serving as a provisional scaffold that guides the migration of SCs, endothelial cells and fibroblasts. Enriching nerve scaffolds with ECM proteins, enables the recreation of a regenerative environment that closely mimics the natural *in vivo* healing process. For instance, incorporating laminin into collagen-based gels stimulates earlier regeneration in short gap injuries (Labrador et al., 1998). However, careful control of concentration and density is crucial in order to modulate the degradation time of the filler, optimizing the regenerating environment for better outcomes. Silicone tubes filled with an extracellular gel containing collagen, laminin, and fibronectin as fillers have been shown to promote successful regeneration across a 10 mm rat sciatic nerve gap, performing significantly better than gels lacking ECM molecules (Chen et al., 2000). More recently, a hollow-channel collagen hydrogel construct coated with ECM proteins (collagen IV, laminin, or fibronectin) was developed to study *in vitro* peripheral nerve regeneration. Among the tested coatings, fibronectin demonstrated the most pronounced effect in enhancing axonal growth and regenerative outcomes (Tusnim et al., 2024).

Numerous studies emphasize the critical role of longitudinal alignment of the ECM components within implanted conduits in order to mimic the natural architecture of the endoneurial tubes in nerve grafts (Gonzalez-Perez et al., 2017; Gonzalez-Perez et al., 2018; Verdú et al., 2002). Pre-alignment of the ECM components, obtained using electrical and magnetic fields, can establish directional cues that guide cell migration and axonal growth, ultimately enhancing the speed and efficacy of nerve regeneration (Rosner et al., 2005). Another effective strategy for enriching hydrogel scaffolds with oriented ECM molecules involves the use of ECM derived from decellularized tissues. This strategy allows preserving the intricate ECM architecture while eliminating cellular components. Preserving the native ECM structure is crucial, as it serves as a biological scaffold guiding cellular behavior in natural tissues (Tamo et al., 2024). For example, hydrogels derived from decellularized porcine nerve tissue have been applied as fillers in nerve conduits, demonstrating the ability to maintain nerve-specific matrix components and growth factors that contribute to enhanced axonal regeneration in nerve gap models (Meder et al., 2021).

The combination of ECM-based scaffolds with cellular transplants has emerged as a promising strategy for peripheral nerve regeneration. Most studies to date have focused on how ECM-based scaffolds or hydrogels support transplanted cell survival, integration, and differentiation, ultimately enhancing nerve repair. To facilitate these processes, various ECM proteins, such as laminin, fibronectin, fibrin, and collagen, have been incorporated into biomaterial scaffolds to promote cell adhesion (Barros et al., 2011; Wang and Sakiyama-Elbert, 2019).

However, relatively few studies have directly investigated the specific role of transplanted cells in synthesizing and depositing ECM components essential for nerve repair. This is a critical distinction, as the endogenous production of ECM by transplanted cells may substantially shape the quality and organization of the regenerative microenvironment (Rao et al., 2022).

For instance, SCs pre-seeded into BD^™^ PuraMatrix^™^ hydrogels have been shown to survive transplantation within nerve conduits and actively promote axonal regeneration (McGrath et al., 2010). These cells not only align along the conduit’s axis but also remodel the matrix by secreting additional ECM molecules. This remodeling supports the formation of aligned cellular structures that closely mimic the natural Bands of Büngner, which are essential for guiding axonal growth in autologous nerve grafts (Gonzalez-Perez et al., 2018; Suri and Schmidt, 2010).

Given the growing interest in bioengineered nerve repair strategies, future research should place greater emphasis on understanding how different transplanted cell types contribute to ECM deposition after transplantation. This remains a relatively underexplored yet potentially vital aspect of optimizing regenerative outcomes in peripheral nerve repair.

### 7.3 Decellularized nerve grafts

Decellularized nerve grafts have been successfully employed in peripheral nerve repair since the preserved ECM provides both structural and functional support useful for nerve fiber regeneration. It is important to clarify that this strategy refers to using the decellularized nerve itself as a conduit, rather than as a filler material, as described in the previous paragraph. Decellularized nerve grafts are produced through a decellularization process that removes immunogenic components while preserving the native ECM and the biomechanical properties of the native nerve. The rationale behind an efficient decellularization protocol is that ECM components are widely conserved across species and are also not adversely immunogenic (Zilic et al., 2015).

This preserved ECM retains essential proteins, glycoproteins, and signaling molecules that support cell adhesion, migration, and differentiation. In decellularized nerve grafts, the ECM acts as a natural scaffold, facilitating the infiltration and alignment of SCs and regenerating axons.

The importance of physical guidance for axons during the regeneration process has been extensively established and current research suggests that the basal lamina and ECM play a key role, creating a permissive environment that supports axonal pathfinding and promotes the maturation of regenerating nerve fibers (Zhao et al., 2011). Lastly, ECM creates functional microvascular networks that support SCs during nerve regeneration (Mahdian et al., 2023).

Various protocols have been developed for preparing decellularized nerve grafts from donor nerves, typically involving a combination of physical, chemical, and enzymatic treatments. To assess their effectiveness, particular attention is given to the preservation of ECM components. Considering the ECM molecules, laminin has been deeply studied due to its pivotal role in promoting myelination (Yu et al., 2023); for this reason, the preservation of laminin is under investigation after decellularization of native nerves in order to evaluate either its preservation or its ability to interact with cells and axons during the regenerative process (Muratori et al., 2024).

Results reported in *in vivo* studies (Contreras et al., 2022; Muratori et al., 2024) allow to consider the possible application of decellularized nerve grafts in the clinical field as an alternative strategy to autograft for repairing nerve injuries.

An important consideration in the development of decellularized nerve grafts is the limited availability of human cadaveric donor nerves, which poses a significant constraint to their widespread clinical application. To address this limitation, xenogeneic tissues have emerged as a promising alternative. Porcine organs share notable anatomical and structural similarities with their human counterparts, and this is especially true for peripheral nerves. Porcine nerves exhibit comparable epineurium, perineurium, and endoneurial microstructure, together with similar ECM organization and composition (Zilic et al., 2015). These characteristics make porcine nerve tissue an attractive and viable candidate for therapeutic applications in peripheral nerve repair (Li R. et al., 2023).

Building on this evidence, many efforts have been directed toward the development of decellularized porcine nerve grafts. An *in vitro* study (Zilic et al., 2016) demonstrated that porcine tibial and peroneal nerves were successfully decellularized using a hypotonic method combined with a low concentration of SDS. Morphological and biochemical analysis demonstrated that the acellular nerves retain their native 3D endoneurial microstructure while eliminating over 95% of the cellular components. The characterization of the ECM revealed the preservation and retention of collagen, laminin, and fibronectin, important ECM components, showing the potential clinical utility of the obtained decellularized porcine nerve as a graft to repair peripheral nerves following injury (Zilic et al., 2016).

A further *in vitro* study (Hopf et al., 2022) compared several decellularization procedures performed on large segments of porcine nerves and highlighted that the enzymatic degradation of chondroitin sulfate proteoglycans reduced the amount of laminin in the graft. Simultaneously, injecting primary adipose-derived mesenchymal stem cells, MSC (hASC), preserves the beneficial features of the ECM on the decellularized nerve, allowing to conclude the good biocompatibility of these scaffolds (Hopf et al., 2022).

Another study (Contreras et al., 2022) assessed the regenerative potential of a novel decellularization protocol applied to human and rat nerves with the aim of repairing rat nerve resection. The decellularization procedure described in this study allowed for the removal of cellular components while preserving the ECM and endoneurial tubules of both rat and human nerves.

Results of this study displayed the fibroblast colonization of the decellularized rat graft at a short-term time point and the presence of regenerated fibers 4 months after repair, suggesting that rat decellularized nerves allowed nerve regeneration through a long gap (Contreras et al., 2022).

Finally, an *in vivo* study (Muratori et al., 2024) introduced a new decellularization protocol, initially used for tendons (Bottagisio et al., 2019), which was applied for the first time to porcine superficial peroneal nerves to produce decellularized nerve grafts. In the context of ECM preservation, the presence of laminin was investigated either on a decellularized porcine nerve graft or on regenerated grafts 4 weeks after implantation. Results showed that laminin was preserved on decellularized porcine grafts, and its presence was also detectable during nerve fiber regeneration in the endoneurial tube, close to regenerated axons, demonstrating the key role of this ECM molecule during the regenerative process.

## 8 Discussion: challenges and future directions for ECM-based strategies in peripheral nerve repair

As highlighted throughout this review and well-documented in literature, the ECM plays a pivotal role in peripheral nerve regeneration by providing both structural support and biochemical cues essential for cellular adhesion, migration, proliferation, and differentiation. Repair strategies that integrate ECM components, such as tubular conduits, hydrogels and decellularized nerve grafts, have demonstrated promising potential in promoting nerve regeneration. While considerable progress has been made in understanding ECM-mediated regenerative mechanisms and in the development of ECM-based therapeutic approaches, several challenges and future directions remain to be explored to fully translate these strategies into consistent and effective clinical applications.

First, while the importance of ECM remodeling during nerve injury and regeneration is well established and its critical role in guiding axonal elongation is recognized, the molecular mechanisms and dynamics of ECM changes during nerve injury and regeneration are not yet fully understood. Further studies are needed to explore how the ECM is reorganized at different stages of nerve repair and how its interaction with other cellular components may influence nerve recovery.

Despite their promising potential, the use of ECM molecules for repairing and enhancing nerve regeneration faces several challenges. One of the most significant is the difficulty in replicating the native ECM’s complex, dynamic composition and mechanical properties within artificial scaffolds. Additionally, the immune response to foreign ECM materials, particularly in xenograft-based approaches, can hinder successful integration and long-term functionality.

Tubular conduits composed of ECM molecules provide a biomimetic scaffold that supports axonal regrowth across nerve gaps by promoting cell adhesion and migration. However, their regenerative capacity is generally limited to short gaps (<3 cm) and they often lack the structural and functional complexity of native nerve tissue. Moreover, only a few ECM components can be used to fabricate conduits due to their mechanical properties. Many studies on these conduits are confined to *in vitro* models, with limited assessment of critical factors such as handling, suturability, and other practical requirements for successful *in vivo* application.

Hydrogels can serve as injectable fillers to enhance tubular conduits for repairing long nerve gaps. When combined with bioactive factors or cells, they create a highly customizable microenvironment that supports cellular infiltration and axonal growth. However, their consistency must balance mechanical stability and permeability to prevent obstruction for regenerating axons, and their degradation rates must align with the timeline of nerve regeneration, avoiding premature loss of structural integrity or prolonged persistence that might impede tissue remodeling.

Regarding decellularized nerve grafts, despite their significant potential to preserve the intricate architecture of the ECM, support cell adhesion and axonal guidance, and minimize immunogenic responses, they also present certain challenges. The decellularization procedures are still time-consuming and labor-intensive, and it can be difficult to preserve important ECM components like laminin, which could reduce the efficacy of the graft.

Another important consideration is the source of ECM molecules used to create nerve devices, conduits or fillers. ECM molecules can be derived from decellularized tissues (Meder et al., 2021) or obtained by culturing various types of cells, including stem cells or somatic cells, as needed (Xu et al., 2024). A notable advantage of using cell-derived ECM is that it can be harvested from the patient’s own tissues, potentially avoiding immune reaction issues when used in nerve conduits. However, ECMs generated from cell cultures are relatively scarce, primarily due to the limited production capacity of the cells, the challenges in maintaining their native structure during *in vitro* conditions, and the variability in ECM composition depending on the cell type, culture medium, and environmental factors (Xu et al., 2024).

In conclusion, despite current limitations, the ECM continues to be a major focus of research due to its numerous beneficial properties and its high potential in the field of regenerative medicine. The ECM not only provides essential structural support but also orchestrates a complex network of biochemical and mechanical signals that regulate cellular behavior and tissue homeostasis. Unlocking the full therapeutic potential of ECM-based strategies will require a deeper understanding of the dynamic and context-specific interactions between ECM components, resident cells, and the surrounding microenvironment. Moreover, the use of innovative bioengineering strategies combining high precision 3D printing techniques (Li et al., 2025) along with ECM-based natural matrices and cell therapies hold significant promise for advancing regenerative medicine. Advances in biomaterials engineering, tissue decellularization techniques, and biofabrication technologies will be instrumental in refining ECM-based therapies and translating them into more effective and reliable clinical applications.
